# Modeling potential cost‐effectiveness of tirzepatide versus lifestyle modification for patients with overweight and obesity

**DOI:** 10.1002/oby.24310

**Published:** 2025-06-13

**Authors:** Meredith M. Hoog, Hong Kan, Kristen A. Deger, Sonja Sorensen, Lisa M. Neff, Jay Patrick Bae, Emily Ruth Hankosky, Madhumita Murphy, Donna Mojdami, Ivan Houisse, Mack S. Harris

**Affiliations:** ^1^ Eli Lilly and Company Indianapolis Indiana USA; ^2^ Evidera Inc. Wilmington North Carolina USA; ^3^ Evidera Budapest Hungary

## Abstract

**Objective:**

Our objective was to model the potential cost‐effectiveness of tirzepatide as an alternative to lifestyle modification (LSM) for the management of obesity and overweight.

**Methods:**

An individual‐level discrete event simulation was implemented in Microsoft Excel linking short‐term outcomes from the SURMOUNT‐1 trial to key obesity‐related complications to estimate costs and health benefits of tirzepatide (5‐mg, 10‐mg, or 15‐mg doses) and LSM over a lifetime time horizon. Treatment‐related changes in cardiometabolic factors were modeled using data from SURMOUNT‐1; the relationship between patient status and risk of obesity complications was obtained from published literature. Modeled complications included cardiovascular events, onset of type 2 diabetes, cancer, osteoarthritis, and sleep apnea. The model simulated 1000 adult patients with overweight or obesity over their lifetimes, applying a 3% annual discount rate to cost and health outcomes. Only direct medical costs were considered.

**Results:**

Tirzepatide 5, 10, and 15 mg provided 0.54, 0.55, and 0.61 additional quality‐adjusted life years (QALYs) and additional costs of $79,288, $70,453, and $75,839 versus LSM, yielding incremental cost‐effectiveness ratios of $146,331, $127,644, and $125,053 per QALY gained, respectively.

**Conclusions:**

The model predicted that all doses of tirzepatide represent cost‐effective alternatives to LSM for management of overweight and obesity at a willingness‐to‐pay threshold of $150,000 per QALY.

**Study Importance**

**What is already known?**

- Obesity is a chronic, progressive disease that is increasingly prevalent in the United States. People with obesity are subject to increased risk of various complications, including cardiovascular events and type 2 diabetes.
- Lifestyle modification (LSM; i.e., diet and exercise) is the current mainstay of treatment for obesity, but even with intensive lifestyle interventions, most individuals are not able to maintain weight loss. Therefore, alternative treatments for obesity are needed.

**What does this study add?**

- Our model, which employs an individual‐level simulation approach with results driven by the full published outcomes from the SURMOUNT‐1 trial (including cardiometabolic factor change), suggests that tirzepatide may be a cost‐effective alternative to LSM based on typical US willingness‐to‐pay thresholds ranging from $100,000 to $150,000 per quality‐adjusted life year.

**How might these results change the direction of research or the focus of clinical practice?**

- Tirzepatide is a first‐in‐class glucose dependent insulinotropic polypeptide and glucagon‐like peptide‐1 receptor agonist approved by the Food and Drug Administration for treatment of type 2 diabetes and obesity. In the SURMOUNT‐1 trial, the mean change in body weight at week 72 with tirzepatide 15 mg was −22.5%, compared with −2.4% with placebo.
- To understand the potential implications of tirzepatide in clinical practice, estimates of its long‐term economic and clinical impacts are needed.

## INTRODUCTION

Obesity is a chronic disease characterized by abnormal levels of body fat that promote adipose tissue dysfunction, resulting in adverse metabolic, biomechanical, and psychosocial health consequences [1, 2]. People with obesity experience increased risk of other diseases compared with people without obesity, including cardiovascular disease (CVD) and type 2 diabetes (T2D), the chief drivers of disability‐adjusted life years (LYs) among people with obesity, according to a 2015 Global Burden of Disease study [3].

The World Health Organization (WHO) first recognized obesity as a global epidemic in 1997 [4]; its current estimated global prevalence is 15% [5]. The prevalence of obesity in the United States is more than double the global average; a 2020 analysis estimated it to be approximately 40% for men and women [6].

Highly effective pharmacotherapy options for obesity are currently limited. The most commonly used intervention is lifestyle modification (LSM; i.e., diet and exercise). Although LSM may lead to meaningful weight reduction, regain is common. In a meta‐analysis of 29 dietary interventions for obesity, by year 5, average weight reduction was just 3% [7].

Tirzepatide is a long‐acting glucose dependent insulinotropic polypeptide and glucagon‐like peptide‐1 receptor agonist approved by the Food and Drug Administration (FDA) for adults with obesity or overweight with weight‐related complications [8]. The SURMOUNT trial program demonstrated tirzepatide's efficacy: In SURMOUNT‐1 (people without T2D at baseline) and SURMOUNT‐2 (people with T2D at baseline), people treated with tirzepatide and LSM lost significantly more weight over 72 weeks than those on LSM alone [8, 9]. Both trials showed promising 72‐week results for improvement in other metabolic factors with tirzepatide.

Clinical and payer decision‐makers may be interested in the long‐term clinical and economic value of tirzepatide. Thus, the objective of this study was to evaluate the cost‐effectiveness of 5‐, 10‐, and 15‐mg doses of tirzepatide versus LSM over patients' lifetimes. The default analysis adopts a US commercial third‐party payer perspective.

## METHODS

### Model overview and structure

A discrete event simulation was developed to project obesity‐related events over a lifetime. The model tracked patients' levels of metabolic factors over time, specifically as follows: body mass index (BMI), waist circumference, high‐density lipoprotein, triglycerides, systolic blood pressure, and fasting plasma glucose. These factors were then used to estimate risk of key obesity‐related complications (see online Supporting Information Methods). The individual‐level simulation captured patient heterogeneity in baseline characteristics and medical history and allowed for flexible and simple tracking of onset timing for multiple complications. The model was implemented in Microsoft Excel (Microsoft Corp.) using the previously described discretely integrated condition event method [10].

**FIGURE 1:**
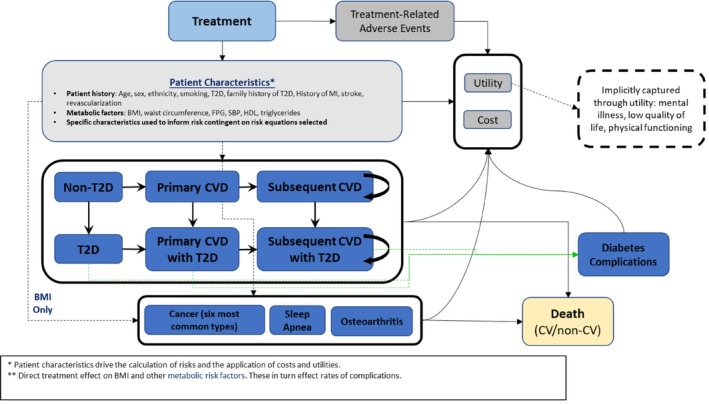
Model diagram. CV, cardiovascular; CVD, cardiovascular disease; FPG, fasting plasma glucose; HDL, high‐density lipoprotein; MI, myocardial infarction; SBP, systolic blood pressure; T2D, type 2 diabetes. [Color figure can be viewed at wileyonlinelibrary.com]

Upon entering the model, a patient profile initiated treatment with tirzepatide 5, 10, or 15 mg or LSM. Identical sets of profiles received each treatment, simulating a perfectly balanced randomized controlled trial. The model then tracked BMI and other metabolic factors affected by treatment; these were updated every 6 months for the first 6 years of the model simulation and every 2 years thereafter and assumed to change linearly across updates. Patients may have developed obesity‐related complications any time in the model based on current metabolic factor levels and demographic criteria (e.g., age, sex, smoking status). Modeled obesity‐related complications included cardiovascular (CV) events, T2D, cancer, sleep apnea, and osteoarthritis (Figure 1). Inclusion of these complications was based on a targeted review of published economic models in obesity. Complications previously shown to be strongly associated with obesity or key long‐term cost drivers were included in the model.

Outcomes tracked by the model included time to onset of obesity‐related complications, costs (total and by category), LYs, quality‐adjusted LYs (QALYs), and incremental cost‐effectiveness ratios (ICER; calculated as $\frac{∆\text{Cost}}{∆\mathrm{LY}}$ or $\frac{∆\text{Cost}}{∆\text{QALY}}$). Cost and health outcomes were discounted by 3% per year, following recommendations from the Second Panel on Cost‐Effectiveness in Health and Medicine [11].

### Treatment phases

**FIGURE 2:**
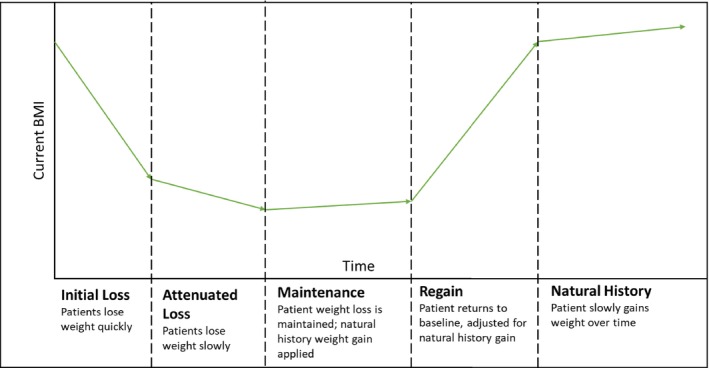
Diagram of BMI trajectory over time. [Color figure can be viewed at wileyonlinelibrary.com]

The model simulated five treatment phases based on weight loss and metabolic factor trajectories observed in clinical trials [8] (Figure 2). First was the *initial loss* phase, wherein patients quickly lost weight and experienced rapid improvement in metabolic factors. Next, patients moved to the *attenuated loss* phase, wherein they continued to lose weight and other metabolic factors improved but more slowly than in the first phase. Third was the *maintenance* phase, wherein patients continued treatment, maintaining most metabolic factor improvement and weight loss achieved in the loss phases, but began regaining a small amount of weight annually (at a rate equivalent to what would be expected if untreated, i.e., according to natural history), until treatment discontinuation. Following discontinuation, patients moved to the *regain* phase, wherein they regained weight until they return to their baseline BMI, plus any weight they would have been expected to gain if untreated, which was assumed to happen over 3 years. Subsequently, patients entered the *natural history* phase, wherein BMI increased over time as patients aged. Additional details on the metabolic factors tracked by the model are in Table S1.

### Inputs

#### Patient characteristics

The National Health and Nutrition Examination Survey (NHANES) informed baseline patient characteristics representative of the US population (Table S2). Profiles were filtered to match FDA eligibility criteria for antiobesity medications (i.e., adults with BMI ≥30 kg/m^2^ and adults with BMI ≥27 kg/m^2^ with at least one obesity‐related complication). Patients with T2D at baseline were excluded.

#### Treatment efficacy and natural history

Tirzepatide and LSM efficacy outcomes were based on efficacy estimands from the SURMOUNT‐1 trial [8]; LSM was based on the placebo arm. Efficacy rather than treatment estimands were used because the impact of treatment discontinuation was considered in the treatment estimand; our model simulated discontinuation and its consequences explicitly. The change in weight and metabolic factors achieved at the end of the loss phases is summarized in Table 1. During the maintenance and natural history phases, patients gained a small amount of weight annually based on natural history data, which vary by sex. Men and women were estimated to gain 0.1447 and 0.1747 kg/m^2^ in BMI per year, respectively [12]. Changes in other metabolic factors during this phase followed patients' BMI; each metabolic factor increased a specific amount per 1‐kg/m^2^ increase in BMI (Tables S3 and S4).

**TABLE 1:** Change in metabolic factors at the end of both loss periods per SURMOUNT‐1, MMRM for efficacy estimand.

Comparator	BMI	WC, cm	HDL	Triglycerides	SBP, mm Hg	FPG, mmol/L
TZP 5 mg, %	−16.0	−12.90	7.00	−24.30	−5.66	−8.07
TZP 10 mg, %	−21.4	−16.90	8.60	−27.00	−6.62	−10.16
TZP 15 mg, %	−22.5	−17.40	8.20	−31.40	−6.18	−11.12
LSM, %	−2.4	−2.98	0.20	−6.30	−0.98	0.94

Abbreviations: FPG, fasting plasma glucose; HDL, high‐density lipoprotein; LSM, lifestyle modification; MMRM, mixed model for repeated measures; SBP, systolic blood pressure; TZP, tirzepatide; WC, waist circumference.

#### Treatment duration and discontinuation

No maximum treatment duration was applied for tirzepatide or LSM, but longitudinal all‐cause discontinuation was modeled using observed trial data (online Supporting Information Methods). Annual discontinuation probabilities for tirzepatide and LSM, based on 72‐week data from SURMOUNT‐1, were 11.2%, 12.2%, 10.6%, and 19.9% annually for tirzepatide 5, 10, and 15 mg and LSM, respectively.

#### Safety

Adverse events (AEs) identified in SURMOUNT‐1 as attributable to either tirzepatide or LSM are generally manageable with treatments that incur no cost to the payer (e.g., nausea and diarrhea, both of which are often manageable with over‐the‐counter medication); thus, these events minimally impacted the model results. Treatment discontinuation due to AEs was captured in the all‐cause discontinuation.

#### Complications and mortality

When possible, complication risk was modeled using published risk equations. Risk equations were sought that included the impact of change in BMI on the event of interest. Such equations were available for CV events and T2D [13, 14]. Risk equations to estimate onset of osteoarthritis, cancer, and sleep apnea that included the impact of BMI were unavailable; therefore, a baseline annual rate of onset was modeled, and the rate was adjusted over time on the basis of patients' current BMI relative to baseline. Because source data only illustrated the relationship between increase in BMI and increase in risk, we conservatively assumed the hazard does not decrease when BMI decreases below baseline (i.e., the rate of these events was only updated when BMI increases).

CVD risk was estimated based on the Framingham 10‐year equations (for primary CV events) [13] and the Long‐Term Intervention with Pravastatin in Ischemic Disease Randomized Controlled Trial (LIPID‐RCT) study (for secondary CV events) [15]. At the time of a CV event, the specific event was assigned, based on data collected by the University of Sheffield that reported the distribution of patients across types of CV events, stratified by primary versus secondary events [16]. CV events captured in the model were stroke, myocardial infarction (MI), and heart failure (HF). Patients may have experienced stroke or MI as primary and/or secondary CV events. Patients may have experienced HF as their primary CV event (potentially in combination with MI or stroke, per the Framingham 10‐year equation) or following a prior MI (i.e., patients with MI are subsequently at risk of developing HF; this risk persists until death). The risk of the latter was informed by Sulo et al. [17]. CV events may be fatal; nonfatal CV events incurred ongoing costs for disease management and a health utility decrement for the event. In addition to mortality associated with CV events, other causes of mortality were captured using US Life Tables [18], adjusted to exclude CV deaths [19].

The onset of T2D was estimated using the Reasons for Geographic and Racial Differences in Stroke (REGARDS) risk equation [20]. After developing T2D, patients received metformin and glimepiride, remaining on treatment for the rest of their lives. Patients with T2D face increased risk of CVD, per the Framingham 10‐year equation, and may also experience diabetes complications, including amputation, diabetic retinopathy, renal disease, and ulcers [21, 22]. The onset of diabetes‐related complications was based on incidence rates collected from patients with T2D; patients with complications incurred additional costs and utility decrements.

The model included cancers shown to have the strongest link with obesity (the top four for each sex), based on a published meta‐analysis [23]. Specifically, these were as follows: colon, kidney, esophageal, pancreatic, endometrial, and breast cancer. Patients were assumed to develop at most one type of cancer during the simulation. Baseline rates of cancer were based on Surveillance, Epidemiology, and End Results (SEER) data [24]. Patients with cancer incurred management costs and a utility decrement. The impact of included cancers on survival was captured using mortality estimates from SEER. Cancer mortality was not excluded from the Life Tables because they do not differentiate across types of cancers. Consequently, the mortality due to cancer may have been overestimated.

Sleep apnea and osteoarthritis were modeled with a baseline rate of developing the condition and an increase in the hazard of onset corresponding to an increase in BMI. Patients with sleep apnea or osteoarthritis incurred management costs and a utility decrement. Baseline rates and hazard ratios associated with each 1 kg/m^2^ of BMI gain for osteoarthritis [25, 26] and sleep apnea [27] were obtained from the literature.

#### Costs

All cost inputs were based on or inflated to 2023 US dollars. A treatment cost of $13,826 per year (Table 2) was applied while patients received tirzepatide. Because the unit cost of tirzepatide was equal across doses, costs were consistent during titration. The cost of LSM considered counseling for obesity, aligned with the SURMOUNT‐1 protocol, estimated to be $1570 in year 1, decreasing to $1515 in subsequent years due to the higher cost of the first in‐person counseling visit [28].

**TABLE 2:** Key cost and utility inputs.

Treatment	Cost (2023 USD)	
TZP (5‐, 10‐, and 15‐mg doses)	$1060/package	
LSM		
First year	$1570/yr	
Subsequent years	$1515/yr	
Concomitant medications		
Lisinopril (SBP >130 mm Hg)	$14/yr	
Metformin and glimepiride (diabetes or FPG >126 mg/dL)	$1087/yr	
Simvastatin (prior CV event or HF)	$23/yr	
**Event**	**Cost (2023 USD)**	
Nonfatal MI		
Hospitalization	$20,196	
First 3 mo of follow‐up	$40,484	
Long‐term annual follow‐up	$6841/yr	
Nonfatal stroke		
Hospitalization	$18,833	
First 3 mo of follow‐up	$28,478	
Long‐term annual follow‐up	$7929/yr	
Nonfatal HF		
Annual follow‐up	$31,592/yr	
CV death	$20,113	
Diabetes complications		
Foot ulcers	$17,887	
Amputation	$29,126	
Diabetic retinopathy	$1008/yr	
Renal disease	$4873/yr	
Osteoarthritis	$6299/yr	
Sleep apnea	$2548/yr	
**Event**	**Utility Multiplier**	
	**Male**	**Female**
Primary MI		
First year	0.8677	0.8953
Subsequent years	0.8917	0.9200
Primary stroke		
First year	0.7629	0.7875
Subsequent years	0.8095	0.8355
Secondary MI		
First year	0.5221	0.5388
Subsequent years	0.8404	0.8678
Secondary stroke		
First year	0.6017	0.6218
Subsequent years	0.7915	0.8174
**Event**	**Utility decrement**	
HF	−0.1034	
Foot ulcer	−0.024	
Amputation	−0.051	
Diabetic retinopathy	−0.058	
Renal disease	−0.04	
Breast cancer	−0.06	
Colon cancer	−0.06	
Endometrial cancer	−0.03	
Kidney cancer	−0.048	
Esophageal cancer	−0.06	
Pancreatic cancer	−0.06	
Osteoarthritis	−0.101	
Sleep apnea	−0.049	

Abbreviations: CV, cardiovascular; FPG, fasting plasma glucose; HF, heart failure; LSM, lifestyle modification; MI, myocardial infarction; SBP, systolic blood pressure; TZP, tirzepatide; USD, US dollars.

Patients received additional treatment based on the complications they experienced in the model. Patients initiated lisinopril if their systolic blood pressure was higher than 130 mm Hg, patients with a previous CV event or HF received simvastatin, and patients with T2D or fasting plasma glucose >26 mg/dL received metformin/glimepiride. Medication costs are shown in Table 2 and Table S5. Table S6 contains details on treatment and event cost sources.

For CV events, costs were linked to the type of event experienced. An acute hospitalization cost was initially applied, and additional follow‐up costs were applied from event onset until death for medical care required after the event (Table 2).

Patients may have experienced multiple diabetes‐related complications, incurring multiple costs (Table S7). Costs for foot ulcer and amputation were applied once at the time of the event. Annual costs for diabetic retinopathy and renal disease were applied until death. Chronic kidney disease stage III management cost was assumed to be representative of the cost of renal disease (Table 2).

Cancer management costs were stratified by age, sex, and first and subsequent years of treatment. Terminal care costs were applied for patients who died of cancer (Tables S8 and S9).

Patients who experienced osteoarthritis or sleep apnea incurred an ongoing management cost until death or the end of the model time horizon, estimated to be $6299 annually [29] and $2548 annually [30], respectively.

#### Health utility

Baseline utility considered patient sex and BMI (Table S10). When patients lost weight, they experienced a utility increment while their BMI was below baseline. This increment was lost when patients returned to BMI equal to or greater than their baseline value. The utility increment was calculated using regression equations described previously [31].

The utility impact of MI and stroke was applied using a multiplicative approach, which considered patient sex and history of CV events [32]. Other utility decrements (for HF [33], osteoarthritis [34], sleep apnea [35], cancer [33, 34, 36], and diabetes‐related complications [37, 38, 39]) were additive: a flat decrease was applied to the utility calculated from baseline (including the impact of patient CVD history). Utility inputs are summarized in Table 2.

### Analyses

In all analyses, 1000 patients (Figures S1 and S2) were simulated over a lifetime horizon, unless otherwise specified. Scenario analyses assessed the impact of different patient populations, risk equations, time horizons, and treatment discontinuation rates (Table 3). Scenario results were presented for a pairwise comparison of tirzepatide 10 mg, as a representative of the three possible tirzepatide doses, against LSM.

**TABLE 3:** Scenario analyses.

Scenario	Description	Rationale
CV event, HF, and T2D only	Simulate only CVD and T2D onset (and their attendant costs and utility effects)	Assess the relative impact of CVD and T2D vs. other complications
Diabetes–San Antonio	Use the San Antonio study equation [43] to simulate onset of diabetes	Multiple risk equations for onset of diabetes available from the literature; assess the impact of their use
Diabetes–Framingham	Use the Framingham study [14] equation to simulate onset of diabetes	
Societal perspective	Include the costs of reduced productivity at work and additional sick time for patients with obesity	Assess the impact of a broader perspective that includes productivity costs in addition to the direct medical costs for patients with obesity
Prediabetes	Include only patients with prediabetes	Assess the impact of restricting TZP treatment to patients afflicted with more severe disease relative to the SURMOUNT‐1 trial inclusion criteria
Obesity class III	Include only patients with BMI of at least 40	
Pessimistic TZP discontinuation	Use TZP discontinuation rate of 50%	Explore the impact of discontinuation rates on the model result
Optimistic TZP discontinuation	Patients on TZP do not discontinue for any reason	
10‐y time horizon	Run simulation for 10 y	Explore the impact of shorter time horizons, which may be of interest to US payers given that patients typically do not keep the same insurer for their entire life
20‐y time horizon	Run simulation for 20 y	
Consider updated White House Guidance on Discount Rates [44]	Run simulation with discount rates set to 2% per annum	Understand the impact of updated White House guidance on appropriate discount rates for CEA, compared with the current 3% guidance
100% smokers at baseline	Simulate a cohort of patients who smoke at baseline	Understand the impact of treating smokers vs. nonsmokers
100% nonsmokers at baseline	Simulate a cohort of patients with no smokers included at baseline	

Abbreviations: CEA, cost‐effectiveness analysis; CVD, cardiovascular disease; HF, heart failure; TZP, tirzepatide; T2D, type 2 diabetes.

Deterministic sensitivity analysis (DSA) was also conducted, changing key parameters to reflect low or high values (informed by 95% confidence interval [CI] values where available, otherwise as ±20% from baseline) to determine drivers of the model results. The DSA also compared tirzepatide 10 mg versus LSM.

Finally, a probabilistic sensitivity analysis was also conducted. Details are available in the online Supporting Information Methods.

## RESULTS

### Base case

In the base‐case analysis, tirzepatide delayed onset of key obesity‐related complications, particularly onset of T2D and primary and secondary CV events. This led to improved discounted LYs and QALYs versus LSM. Specifically, 0.09, 0.09, and 0.10 LYs and 0.54, 0.55, and 0.61 QALYs were gained with tirzepatide 5, 10, and 15 mg, respectively. The additional discounted costs of tirzepatide over LSM were $79,288, $70,453, and $75,839 for the 5‐, 10‐, and 15‐mg doses, respectively. The driver of additional costs was the increased treatment cost of tirzepatide relative to LSM, although this increase was mitigated by reduced incidence of obesity‐related complications. The incremental cost of tirzepatide 10 mg versus LSM was lower than the incremental costs of tirzepatide 5 mg and 15 mg versus LSM due to the higher discontinuation rate observed for the 10‐mg dose in SURMOUNT‐1. The resulting ICER versus LSM was $146,331, $127,644, and $125,053 for tirzepatide 5, 10, and 15 mg, respectively (Table 4).

**TABLE 4:** Discounted results.

Absolute results	TZP 5 mg	TZP 10 mg	TZP 15 mg	LSM
Mean time to key events, yr				
Onset of T2D	18.61	18.96	18.91	17.23
Primary MI	20.21	20.21	20.33	19.81
Primary stroke	22.65	22.47	22.75	21.52
Secondary MI	24.88	24.97	25.15	24.71
Secondary stroke	23.01	22.91	23.47	20.14
Key cost outcomes, $				
Treatment costs	86,480	77,544	82,947	5538
CV event and HF costs	20,939	20,821	20,696	21,615
Cancer management and terminal costs	9575	9795	9939	9687
Diabetes management and complication costs	6512	6379	6323	7142
Osteoarthritis costs	6979	6988	6983	6915
Sleep apnea costs	19,572	19,642	19,652	19,600
Total costs	157,110	148,274	153,661	77,821
Health outcomes				
LYs	19.80	19.80	19.81	19.71
QALYs	15.06	15.07	15.12	14.51

Incremental results	TZP 5 mg vs. LSM	TZP 10 mg vs. LSM	TZP 15 mg vs. LSM
Incremental costs, $	79,288	70,453	75,839
Incremental LYs	0.09	0.09	0.10
Incremental QALYs	0.54	0.55	0.61
ICER/QALY, $	146,331	127,644	125,053

Abbreviations: CV, cardiovascular; HF, heart failure; ICER, incremental cost‐effectiveness ratio; LSM, lifestyle modification; LY, life year; MI, myocardial infarction; QALY, quality‐adjusted life year; TZP, tirzepatide; T2D, type 2 diabetes.

### Scenarios

Scenario analyses were consistent with the base‐case analysis; tirzepatide delivered increased LYs and QALYs at additional costs relative to LSM (Table 5). Only two analyses produced an ICER above the commonly used US willingness‐to‐pay threshold of $150,000 per QALY.

**TABLE 5:** Scenario results: discounted (all results are TZP 10 mg vs. LSM).

Scenario	Incremental			ICER/QALY, $
	Costs, $	LY	QALY	
Base case	70,453	0.09	0.55	127,644
CVD and T2D only	71,096	0.09	0.55	128,461
Diabetes–San Antonio	66,530	0.12	0.59	112,013
Diabetes–Framingham	63,384	0.17	0.65	97,391
Societal perspective	63,954	0.09	0.55	115,870
Prediabetes	66,033	0.09	0.51	129,347
Obesity class III	72,810	0.07	0.64	113,640
Pessimistic TZP discontinuation	15,779	0.00	0.09	172,950
Optimistic TZP discontinuation	256,482	0.39	2.14	120,130
10‐y time horizon	57,739	0.02	0.38	150,887
20‐y time horizon	68,139	0.05	0.51	133,228
2% discount rate for cost and health outcomes	74,426	0.11	0.60	124,906
100% smokers at baseline	69,315	0.10	0.61	114,396
100% nonsmokers at baseline	69,065	0.04	0.52	132,342

Abbreviations: CVD, cardiovascular disease; ICER, incremental cost‐effectiveness ratio; LSM, lifestyle modification; LY, life year; QALY, quality‐adjusted life year; TZP, tirzepatide; T2D, type 2 diabetes.

Results accounting only for the impact on CVD and T2D produced similar incremental QALYs, LYs, and costs to the base case, suggesting that improvement in CVD and T2D outcomes is a driver of the economic value of tirzepatide, with the remaining complications having more marginal impact. Among T2D risk equations, both alternates decreased the ICER relative to the base‐case result, suggesting that the use of REGARDS is conservative. The Framingham analysis yielded the largest difference in patients developing T2D, producing the lowest ICER of $97,391 per QALY versus LSM. Accounting for indirect costs led to improved results for tirzepatide, reducing incremental cost to $63,954, yielding an ICER of $115,870 per QALY. Scenario results suggested that targeting tirzepatide for patients with at least class III obesity would be more cost‐effective, with an estimated ICER of $113,640 for these patients. Extreme assumptions on discontinuation rates had a notable impact on both cost and QALY outcomes. An optimistic scenario in which patients remained on tirzepatide indefinitely showed a similar ICER to the base case, with significantly higher incremental costs and QALYs, indicating a stable and proportional relationship between ongoing treatment costs and QALYs gained in the long term. In the pessimistic scenario in which all patients discontinued treatment quickly, the result was significantly lower incremental QALYs and costs, resulting in an increased ICER. The accelerated discontinuation rate is unlikely to occur in the real world given that this scenario causes most patients to discontinue treatment prior to realizing its maximum effect. The increased ICER observed with restricted time horizons supported the hypothesis that the economic benefits of tirzepatide are best captured over a lifetime.

### Sensitivity

**FIGURE 3:**
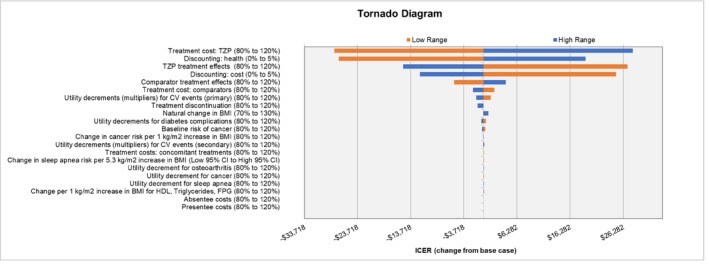
DSA tornado diagram. DSA, deterministic sensitivity analysis; CV, cardiovascular; FPG, fasting plasma glucose; HDL, high‐density lipoprotein; ICER, incremental cost‐effectiveness ratios; TZP, tirzepatide. [Color figure can be viewed at wileyonlinelibrary.com]

The DSA showed that key result drivers are the cost of tirzepatide, discount rates for costs and health, the clinical efficacy of tirzepatide, and the clinical efficacy of LSM (Figure 3).

The probabilistic sensitivity analysis showed that tirzepatide 10 mg would have a 23% probability of cost‐effectiveness at a willingness to pay of $100,000, increasing to 57% at a willingness to pay of $150,000.

## DISCUSSION

The Institute for Clinical and Economic Review considers medications to be cost‐effective at an ICER between $100,000 and $150,000 per QALY [40]. In this model, all doses of tirzepatide were cost‐effective alternatives to LSM, with the ICER falling below the upper bound of that range.

In this analysis, each individual tirzepatide dose was directly compared with LSM for the sake of transparency, rather than being presented as a fully incremental analysis. In practice, all three doses are included in the approved prescribing information, each with the same unit cost. Patients are likely to titrate through multiple dose levels, ultimately stabilizing at the maximum tolerated dose. Consequently, no formulary should cover only a single dose of tirzepatide, and it is unlikely medical decision‐makers will face a scenario where they must choose between prescribing two doses of tirzepatide. The DSA illustrated that result drivers include discounting rates, the cost of tirzepatide, and the clinical efficacy of tirzepatide and LSM. As the cost of tirzepatide is unlikely to be subject to uncertainty and the 72‐week efficacy and discontinuation rates were explored in the SURMOUNT‐1 trial, this suggests that future research should focus on developing long‐term estimates of tirzepatide's efficacy and discontinuation rates.

Scenario analysis indicated that a key driver of the economic value of tirzepatide is the reduction in the number of patients developing CVD and T2D and that the choice of risk equation to model those developing T2D in the base case is conservative. An optimistic scenario in which patients did not discontinue tirzepatide for any reason produced an ICER similar to the base case, whereas a scenario in which patients discontinued tirzepatide quickly yielded a notably higher ICER than the base case, suggesting long‐term adherence is an important treatment goal. Inclusion of societal perspective costs and restricting the patient population to those that have more severe obesity (class III) led to improved cost‐effectiveness results. The other scenario that yielded an ICER above $150,000 per QALY was the 10‐year time horizon scenario, which was slightly above this threshold. The short time horizon is insufficient to fully recognize the benefit of tirzepatide in terms of reduced complication rates.

To provide context, we compared our results with recently published cost‐effectiveness analyses in obesity, particularly the model assessing semaglutide 2.4 mg published by Kim et al. [41] and the analysis published by the Institute for Clinical and Economic Review [42]. Our model estimated higher incremental costs and QALYs for tirzepatide compared with the model by Kim et al., which predicted that semaglutide 2.4 mg would yield incremental QALYs of 0.18 and incremental costs of $22,138 (ICER: $122,549/QALY) versus LSM. An important difference between that analysis and ours is treatment duration. In Kim et al., the model assumed a maximum treatment duration of 2 years for semaglutide, whereas our model has no fixed treatment duration. We hypothesize that application of similar treatment rules in the semaglutide model could produce incremental costs and QALYs more consistent with our analysis.

In the recently published evidence report for antiobesity medications by the Institute for Clinical and Economic Review [42], Drug X (i.e., tirzepatide, based on top‐level findings of the SURMOUNT‐1 trial and assuming an annual price of $13,618) was estimated to yield 0.48 LYs and 1.30 QALYs at an incremental cost of $188,000 over LSM. Our analysis has two important differences: first, we leveraged published SURMOUNT‐1 data, which estimated a BMI decrease greater than the 17.8% used by the Institute for Clinical and Economic Review; second, our model captures longitudinal discontinuation. Specifically, their model only considered discontinuations due to AEs rather than discontinuation due to any cause as in our model. Further, their model did not consider discontinuations over time; instead, the impact of discontinuation was applied only during the first cycle. The incremental costs and QALYs for the optimistic discontinuation scenario in our model (Table 5) are more similar to their result. The same limitations around discontinuation and its application apply to the Institute for Clinical and Economic Review analysis of semaglutide, which in their model was found to yield higher LYs, QALYs, and incremental costs compared with LSM than any dose of tirzepatide in our analyses.

The results of this analysis should be interpreted with respect to its limitations, which relate primarily to the availability of data for the target population, the long‐term uncertainty, and the model's “indirect” approach to complication estimation.

In terms of data availability, the model uses risk equations developed using populations including patients with and without obesity. Although BMI and other metabolic factors were included as risk factors, it is possible that the impact of these covariates may differ in a population composed exclusively of individuals with obesity. Furthermore, detailed risk equations were unavailable for sleep apnea, osteoarthritis, and cancer. To assess the impact of the T2D risk equations, alternates were explored in scenario analyses. Alternates to the CVD risk equations, Framingham 10‐year and LIPID‐RCT, suitable for the model could not be identified, so the impact of their use could not be assessed. Additionally, long‐term treatment adherence to tirzepatide is not currently known. The discontinuation rates used in this model were based on trial data, which captured discontinuation for reasons related to patients' overall experience of treatment but did not capture discontinuation for reasons related to access. Future research could incorporate real‐world evidence on adherence into the model once such data become available.

Long‐term uncertainty in the model is driven primarily by the availability of data to inform the treatment effect of tirzepatide from SURMOUNT‐1. Although trial data followed patients up to 72 weeks, our model sought to understand the lifetime BMI trajectory of simulated patients. Therefore, we supplemented the data with several key assumptions, namely the following: BMI loss persists until patients stop treatment, excepting natural history growth, and patients gain a small amount of weight each year when they stop active treatment. The model could be improved with evidence indicating how the treatment effect of tirzepatide persists in the long term for patients who continue to stay on treatment.

A third limitation of this model is the use of an “indirect” approach to treatment effect, in which the impact on complication development is mediated via the change in BMI and other metabolic characteristics. It would be preferable to instead directly capture the impact of tirzepatide on CV events; to that end, a CV outcomes trial for tirzepatide is currently in progress.

Despite the noted limitations, the model's structure lends several strengths to the analysis. First, the individual‐level approach is well suited to capture patient heterogeneity, in terms of complication status and metabolic factor levels at baseline. Additionally, the individual‐level approach allows for flexibility to specify population subsets using multiple selection criteria. The individual‐level approach simplifies the tracking of comorbidities compared with commonly used state‐based approaches. In state‐based models, all health states must be defined in such a way that they are mutually exclusive, which necessitates the inclusion of many health states; this may in turn increase the difficulty of implementing and verifying the model [41]. In comparison, an individual‐level model may simply “enable” the presence of a complication for a given patient and estimate consequences based on the new complication plus any previously existing complications. Finally, compared with previous models, our model allows for flexibility in the approach to treatment discontinuation: It can capture longitudinal discontinuation over time and its impact on both costs and complication risks, which were not considered in either the Kim et al. model or the Institute for Clinical and Economic Review model. A list of model assumptions is presented in Table S11.

## CONCLUSION

According to this simulation, tirzepatide may be considered a cost‐effective alternative to LSM for the treatment of obesity, with our model predicting increased LYs and QALYs over LSM with all doses and yielding an ICER below $150,000 per QALY.

## AUTHOR CONTRIBUTIONS

Ivan Houisse and Mack S. Harris implemented the model. Kristen A. Deger, Benjamin White, and Mack S. Harris conducted literature searches. All authors contributed to data interpretation and manuscript writing and had final approval of the submitted and published versions.

## FUNDING INFORMATION

The study was funded by Eli Lilly and Company. Evidera contributors only received their standard Evidera salaries, with no specific grants or remuneration directly from Eli Lilly.

## CONFLICT OF INTEREST STATEMENT

Kristen A. Deger, Sonja Sorensen, Ivan Houisse, and Mack S. Harris are employees of Evidera Inc., which received consulting fees from Eli Lilly and Company to support the research described in this paper. Meredith M. Hoog, Hong Kan, Jay Patrick Bae, Emily Ruth Hankosky, Donna Mojdami, Madhumita Murphy, and Lisa M. Neff are employees of Eli Lilly and Company and may hold shares and/or stock options in the company.

## Supporting information


**Data S1.** Supporting Information.

## Data Availability

The data that supports the findings of this study are available in the online Supporting Information of this article.
