# Supplementary material for: Modeling potential cost‐effectiveness of tirzepatide versus lifestyle modification for patients with overweight and obesity
Source: Obesity (Silver Spring). 2025 Jun 13;33(7):1297–308. doi: 10.1002/oby.24310 (PMC12210105; doi:10.1002/oby.24310)
Supplement: Supplementary file 1 — Data S1. Supporting Information. [file OBY-33-1297-s001.docx]

# Supplementary Appendix

## Methods

### Scheduling and Updating Time to Event Estimates

To prevent over-prediction of risk, while allowing the model to run efficiently and capture all significant changes in cardiometabolic factors, the model updates time to event estimates at the following points:

1. When a patient completes one treatment phase and moves to another (i.e., end of initial loss, end of attenuated loss, end of maintenance, and end of regain phases)
2. Triggered by the onset of other events:
   1. Cardiovascular (CV) risks are updated after the onset of diabetes,
   2. Risk of a secondary CV event is estimated after a primary CV event occurs,
3. Regularly scheduled model updates: every 0.5 years for the first 6 years and every 2 years thereafter.

CV events, the onset of diabetes, diabetes-related complications, obstructive sleep apnea (OSA), osteoarthritis, and cancer are scheduled using a random number draw and an exponential time-to-event distribution with the calculated event rate as the rate parameter. Thus, a random number of 0.5 would yield the estimated median event time given the predicted rate. In cases where an event was predicted but the event time is subsequently updated to account for a change in cardiometabolic parameters, the update calculations account for the patient’s “spent luck”. Specifically, the random number draw for the new survival estimate is multiplied by the probability of the patient surviving to the current time without an event.

The model estimates when events will occur, schedules them all at once, and then interprets the event outcomes in the order they are scheduled to occur. Because the timing of non-CV death is scheduled only once at model entry, events may be prevented if scheduled to occur after non-CV death, or, conversely, new events may occur if deteriorating patient characteristics cause them to be scheduled prior to non-CV death.

Consider the example of a patient who is initially scheduled to have diabetes onset at year 2, a primary CV event at year 5, and non-CV death at year 7. In one scenario this hypothetical patient would respond very well to treatment, and as a result, their updated time to diabetes is now scheduled for year 6 and their estimated time of a primary CV event is year 10. The model reaches year 6 and updates the estimated time to primary CV event, now year 8. However, when the model reaches year 7, the non-CV death takes effect, and the primary CV event would then never occur.

Cumulative incidence of mortality, diabetes, and CV events are presented in Figures S3, 34, and S5, respectively.

### Discontinuation

Discontinuation is scheduled in the same manner any other event, i.e., interpreting the annual probability as the rate parameter in an exponential distribution, and scheduling the discontinuation time using a random number. Patients remain on treatment until the discontinuation event occurs. Discontinuation is scheduled at the time that the patient begins treatment, time zero in the model. The main consequences of treatment discontinuation captured in the model are:

1. The patient stops incurring treatment costs.
2. The patient moves to the “regain” phase of treatment, returning to their baseline BMI, plus any natural history-related weight gain, three years after discontinuation. Patients are assumed to regain weight linearly.

### Probabilistic Sensitivity Analysis

In the probabilistic sensitivity analysis (PSA), all parameters are varied according to estimated uncertainty. In cases where uncertainty data was not available, a standard error was calculated by assuming that the upper and lower 95% confidence interval were 10% higher and 10% lower than the mean value, respectively. The distributions used to vary parameters followed standard health economic practices. For example, a beta distribution is used for utilities and probabilities and other values that are bounded between 0 and 1, the gamma distribution is used for costs, and the lognormal distribution is used for rates and hazard ratios. In cases where no specific distribution is recommended, a normal distribution was used.

The analysis was run using tirzepatide 10mg as the representative dose. 1,000 simulations were run, although stability plotting shows that a stable result is achieved by approximately replication 200 for LY and QALY, and approximately replication 500 for costs (Figure S6, Figure S7, and Figure S8 for incremental LY, incremental QALY, and incremental costs, respectively). The cost-effectiveness (CE) plane is shown in Figure S9 and cost-effectiveness acceptability curve (CEAC) is in Figure S10.

Within the PSA findings, 98% of iterations showed tirzepatide 10mg to be more effective and more costly than LSM, yielding an ICER result in the first quadrant of the CE plane (positive Δ QALY and positive Δ cost). The PSA also showed that incremental cost and incremental QALY outcomes were not strongly correlated: incremental costs for most simulations fell within a relatively narrow band from approximately $80,000 to $60,000, while incremental QALY results were more widely distributed and crossed zero. These ranged from a gain of approximately 1.2 QALYs in the most optimistic simulation, to a loss of approximately 0.3 QALYs in the most pessimistic simulation.

The CEAC shows that as the WTP threshold increases above $75,000, the probability that tirzepatide 10mg can be considered cost-effective rises rapidly. Specifically, it had a 23% probability of being found cost effective at a WTP threshold of $100,000; nearly tripling to 57% at a WTP threshold of $150,000.

## Tables

Table S1. Metabolic Factors Included in the Model

| Metabolic Factor | Use in the Model |
| --- | --- |
| BMI | A coefficient in the risk equations predicting diabetes and CV events. Driver of the risk of sleep apnea, osteoarthritis, and cancer. Impacts patient health utility. |
| WC | Influences the risk of diabetes in the scenario analysis that uses the Framingham Offspring Study risk equation. |
| HDL | Influences the risk of diabetes (for all risk equations). |
| Triglyceride levels | Influences the risk of diabetes (in the base case using the REGARDS study risk equation and scenario analysis using the Framingham Offspring study risk equations). |
| SBP | Influences the risk of diabetes (in the base case and in scenario analysis using the San Antonio study risk equation). Influences the risk of primary CV events. Patients with an SBP greater than 130 mmHg begin treatment with antihypertensives. |
| FPG | Influences the risk of diabetes (in scenario analysis using the San Antonio Study risk equation). Patients with FPG of at least 126 mg/dL initiate treatment with metformin and glimepiride. |

Abbreviations: BMI = body mass index; CV = cardiovascular; FPG = fasting plasma glucose; HDL = high-density lipoprotein; SBP = systolic blood pressure; WC = waist circumference

Table S2. Base Case Population Characteristics*

| Characteristic | Mean | SD | Min | Max |
| --- | --- | --- | --- | --- |
| Demographics | | | | |
| % male | 47% |  |  |  |
| Age (years) | 45.68 | 16.76 | 18.00 | 80.00 |
| Metabolic Factors | | | | |
| BMI (kg/m^2^) | 35.08 | 6.87 | 27.00 | 92.30 |
| Waist circumference (cm) | 112.30 | 13.08 | 77.20 | 173.10 |
| HDL (mg/dL) | 50.39 | 13.43 | 21.00 | 151.00 |
| Triglycerides (mg/dL) | 136.13 | 103.67 | 33.00 | 2,923.00 |
| SBP (mm Hg) | 121.77 | 18.18 | 78.00 | 213.00 |
| FPG (mmol/L) | 102.83 | 9.98 | 63.00 | 126.00 |
| Medical History | | | | |
| Prior stroke | 4% |  |  |  |
| Prior MI | 3% |  |  |  |
| Prior revascularization | 4% |  |  |  |
| Prior CHF | 2% |  |  |  |
| Pre-diabetes | 17% |  |  |  |

NHANES data represents that collected from 2017 – 2020; i.e., the most complete pre-pandemic dataset.

Abbreviations: BMI = body mass index; CHF = congestive heard failure; FPG = fasting plasma glucose; HDL = high-density lipoprotein; MI = myocardial infarction; SBP = systolic blood pressure
*SD, min, and max are shown for continuous variables only.

Table S3. Natural History Changes in HDL, Triglycerides, and FPG

| Metabolic Factor | Change per 1 kg/m^2^ increase in BMI | Source |
| --- | --- | --- |
| HDL | -0.25 mg/dL | Shamai 2012^1^ |
| Triglycerides | 1.00 mg/dL |  |
| FPG | 0.5 mg/dL | Assumption |

Abbreviations: BMI = body mass index; FPG = fasting plasma glucose; HDL = high-density lipoprotein

Table S4. Natural History Changes in SBP

| Sex | BMI (kg/m^2^) | SBP change (mmHg) per 1kg/m^2^ increase in BMI | | | Source |
| --- | --- | --- | --- | --- | --- |
|  |  | *Treated with anti-hypertensives* | *Not treated with anti-hypertensives* | |  |
| Men | 20 to ≤25 | 0.76 | | 0.74 | Adler 2015^2^ |
|  | >25 to ≤30 | 0.54 | | 0.60 |  |
|  | >30 | 0.34 | | 0.48 |  |
| Women | 20 to ≤25 | 1.52 | | 1.26 |  |
|  | >25 to ≤30 | 0.96 | | 0.80 |  |
|  | >30 | 0.40 | | 0.34 |  |

Abbreviations: BMI = body mass index; SBP = systolic blood pressure

Table S5. Concomitant Medications

| Drug | Pack Cost | Pill Size | Pill Count | Dose | Complication Managed |
| --- | --- | --- | --- | --- | --- |
| Simvastatin | $2.84 | 20 mg | 90 pills | 20mg / day | Dyslipidemia |
| Lisinopril | $3.92 | 40 mg | 100 pills | 40mg / day | Hypertension |
| Metformin | $82.96 | 1000 mg | 60 pills | 1000mg / day | Dysglycemia |
| Glimepiride | $10.70 | 4 mg | 100 pills | 4 mg / day | Dysglycemia |

Table S6. Treatment and Event Cost Sources

| Cost Category | Cost* | Source |
| --- | --- | --- |
| Intervention and other drug unit costs | Unit cost of tirzepatide and concomitant medications | RED BOOK®^3^ |
|  | Unit cost of LSM (in office PCP visits - $158 for initial and $104 for follow-up) | InHealth Professional Services 2020^4^ |
| CV event costs | One-time cost of CV hospitalization due to non-fatal MI, stroke, or CV death | CMS^5^ |
|  | Ongoing follow-up costs related to MI and stroke | Bonafede 2015^6^ |
|  | Ongoing follow-up costs related to HF | Urbich 2020^7^ |
| Diabetes complications | Foot ulcer (one-time) | HCUP^8^ |
|  | Amputation (one-time) |  |
|  | Diabetic retinopathy (ongoing) | Rein 2006^9^ |
|  | Chronic kidney disease (ongoing) | Honeycutt 2013^10^ |

*Costs are inflated to 2023 USD

Abbreviations: CV = cardiovascular; HF = heart failure; LSM = lifestyle modification; MI = myocardial infarction; PCP = primary care practitioner

Table S7. Annual Rate of Onset for Diabetes Complications

| Complication | Incidence | Source |
| --- | --- | --- |
| Foot Ulcers* | 1.13% | NIH Diabetes in America, 3^rd^ Edition Report, Chapter 20^11^ |
| Amputation | 0.05% | An 2021^12^ |
| Diabetic Retinopathy | 0.12% |  |
| Renal Disease | 1.33% |  |

Table S8. Cancer Management Costs

|  | Male | | | | Female | | | |
| --- | --- | --- | --- | --- | --- | --- | --- | --- |
|  | **Age < 65** | | **Age ≥ 65** | | **Age < 65** | | **Age ≥ 65** | |
|  | **First year cost** | **Subsequent year cost*** | **First year cost** | **Subsequent year cost*** | **First year cost** | **Subsequent year cost*** | **First year cost** | **Subsequent year cost*** |
| Breast cancer | $0 | $0 | $0 | $0 | $38,554 | $3,073 | $32,129 | $3,073 |
| Colorectal cancer | $86,559 | $4,398 | $72,133 | $6,397 | $85,750 | $4,398 | $71,458 | $4,398 |
| Endometrial cancer | $0 | $0 | $0 | $0 | $55,929 | $8,157 | $55,929 | $8,157 |
| Kidney cancer | $64,109 | $8,378 | $53,425 | $8,378 | $64,149 | $8,708 | $53,457 | $8,708 |
| Esophageal cancer | $133,356 | $8,980 | $111,129 | $8,980 | $132,871 | $9,541 | $110,725 | $9,541 |
| Pancreatic cancer | $157,196 | $16,285 | $130,996 | $16,285 | $156,142 | $12,073 | $130,119 | $12,073 |

**Source: Mariotto 2011^13^; inflated to 2023 USD**

Table S9. Cancer Terminal Care Costs

|  | Male | Female |
| --- | --- | --- |
| Breast cancer | $0 | $131,263 |
| Colorectal cancer | $178,909 | $176,502 |
| Endometrial cancer | $0 | $133,349 |
| Kidney cancer | $163,060 | $154,208 |
| Esophageal cancer | $216,657 | $217,765 |
| Pancreatic cancer | $236,221 | $229,591 |

**Source: Mariotto 2011^13^; inflated to 2023 USD**

Table S10. Baseline Utility by Age and BMI

| BMI Level | Ages 18 – 30 | Ages 31 – 40 | Ages 41 – 50 | Ages 51 – 60 | Ages 61 - 70 | Age ≥ 71 |
| --- | --- | --- | --- | --- | --- | --- |
| < 30 | 0.91 | 0.89 | 0.86 | 0.83 | 0.81 | 0.79 |
| 30 – 34.9 | 0.89 | 0.86 | 0.82 | 0.80 | 0.79 | 0.76 |
| 35 – 39.9 | 0.88 | 0.83 | 0.79 | 0.77 | 0.76 | 0.74 |
| 40 – 49.9 | 0.84 | 0.82 | 0.75 | 0.73 | 0.71 | 0.69 |
| ≥ 50 | 0.80 | 0.77 | 0.70 | 0.69 | 0.66 | 0.66 |

**Source: Alsumali 2018^14^**

Abbreviations: BMI = body mass index

Table S11. Important Model Assumptions

| Category | Assumption |
| --- | --- |
| BMI and Other Metabolic Factors | BMI and other metabolic factors are assumed to change at a constant rate (linearly) between updates. |
|  | Treatment effects on other (non-BMI) metabolic factors in the initial loss, attenuated loss, maintenance, and regain phases are assumed to be independent of BMI change; inputs should be considered carefully to minimize double counting of effects. |
|  | The model draws the expected metabolic factor change from a normal distribution centered around the mean predicted effect. |
|  | In the natural history phase (post treatment), non-BMI metabolic factors are assumed to change in proportion to natural changes in BMI. |
|  | In the initial loss, attenuated loss, maintenance, and regain phases, a patient's waist circumference is considered to be the minimum of:  (1) baseline waist circumference plus any treatment effect  (2) the calculated value based on a prediction equation.  In the natural history phase (post treatment), the calculated value based on the prediction equation is used. |
| Complications | Obesity is a multifaceted disease and a primary risk factor for a wide range of health conditions and comorbidities. It is not feasible to capture all associated complications in the model, thus the most common complications from previously published models were prioritized. |
|  | As a simplifying assumption, patients can have only one type of cancer at a time. Once a patient contracts one type of cancer, cancer risk calculations are turned off and they cannot experience other types of cancer simultaneously. |
|  | Owing to data limitations, the onset of complications other than CV events and T2D are linked to BMI alone. |
|  | The model assumes no delays in diagnosis or treatment of diabetes following its onset in a given patient. |
|  | Breast cancer applies only to post-menopausal women; menopause is assumed to begin at age 50. |
| Risk Equations | CVD and T2D risks are initially set at baseline and then only recalculated at the end of each treatment phase in the first phase of the model (i.e., before switch to the secondary cycle length). They are recalculated at each BMI update cycle in the second phase of the model (as well as at changes of treatment phase if any change of treatment phase occurs during this second phase of the model). The baseline age is considered for CVD and diabetes risk re-calculations over time. Keeping baseline age the same allows the model to more clearly articulate differences in treatment effect. |
|  | It is assumed that the risk equations, which were developed in populations which had not been selected for obesity, can be applied to populations comprised exclusively of patients with obesity. |
|  | Non-CV mortality was adjusted to exclude CV-related death but not cancer mortality. Cancer mortality could not be adjusted for as the model captures only a few key cancers and the type of cancer is not distinguished within the non-CV mortality source. Therefore, it may be the case that the model is overestimating cancer related mortality. |
|  | The cerebrovascular events estimated by the Framingham 10-year risk equation include both fatal and non-fatal events. When an event is fatal, the patient's death is captured at that time in the model. Non-CV mortality is linked to US lifetable estimates. |
| Costs | Long-term follow-up costs for MI and stroke events are the costs applied post-3 months following the event, and are applied for the remainder of life in the model. These costs are not applied to patients entering the model with a history of MI/stroke (unless they experience another MI/stroke later during the simulation). |
|  | HF is assumed not to incur an acute cost at the time of onset, but does initiate an ongoing follow-up cost. |
|  | If a patient has had multiple CV events, the maximum cost attributed to those events will be applied. |
|  | Drug wastage (i.e., drug left over in the pack/vial that could not be used by the patient) is not accounted for. |
| Utility | Patients may experience a utility increment from BMI loss only when their BMI is lower than their baseline value. Once patients are at or above their baseline BMI, they lose any BMI-related utility increments and revert to the baseline utility for their age and BMI category. |
|  | Long-term utility decrements for MI and stroke events are those applied post-1 year following the event, and are applied for the remainder of life in the model. For patients entering the model with a history of MI/stroke, the long-term utility decrements for primary MI and/or primary stroke (as relevant) are applied from model start. |
|  | If a patient experiences both MI(s) and stroke(s), the product of the utility decrements (multipliers) attributed to each of MI and stroke will be applied. |
|  | Use of the trial-based generic HRQoL measures in the future will require re-assessment of any potential assumptions or limitations (e.g., osteoarthritis effects may already be captured in the physical functioning component of these measures). |

Abbreviations: BMI = body mass index; CV = cardiovascular; CVD = cardiovascular disease; HF = heart failure; HRQoL = health-related quality of life; MI = myocardial infarction; T2D = type 2 diabetes

## Figures

Figure S1. Stability Plot: Incremental QALYs, TZP vs. LSM
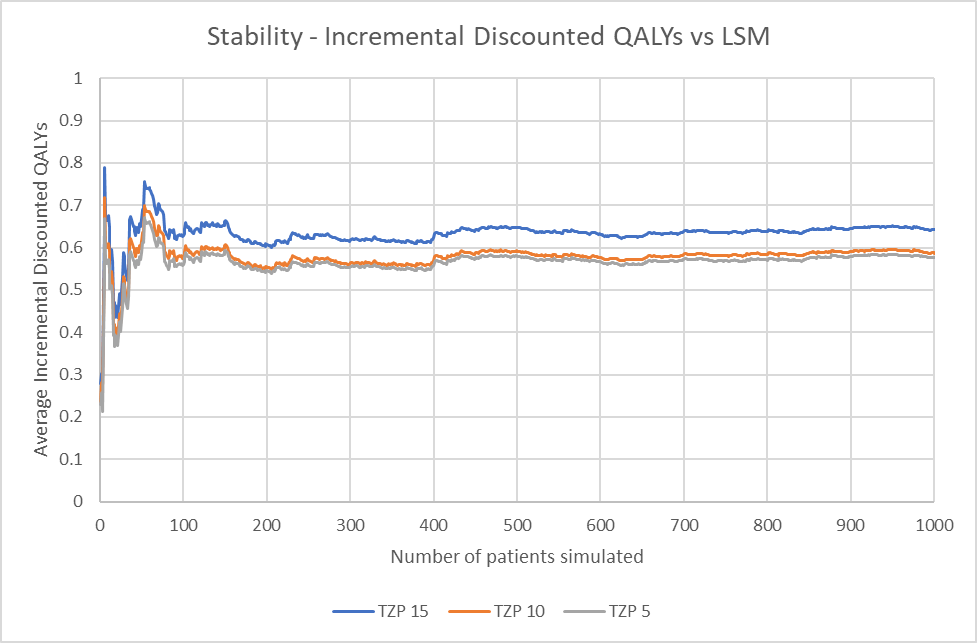


Stability plots show the average incremental outcome on the y-axis, given the number of patients simulated on the x-axis. They are used to assess whether a sufficient number of replications have been run to achieve a stable result, indicated by the presence of a flat line.

*Figure S2. Stability Plot: Incremental Costs, TZP vs. LSM*
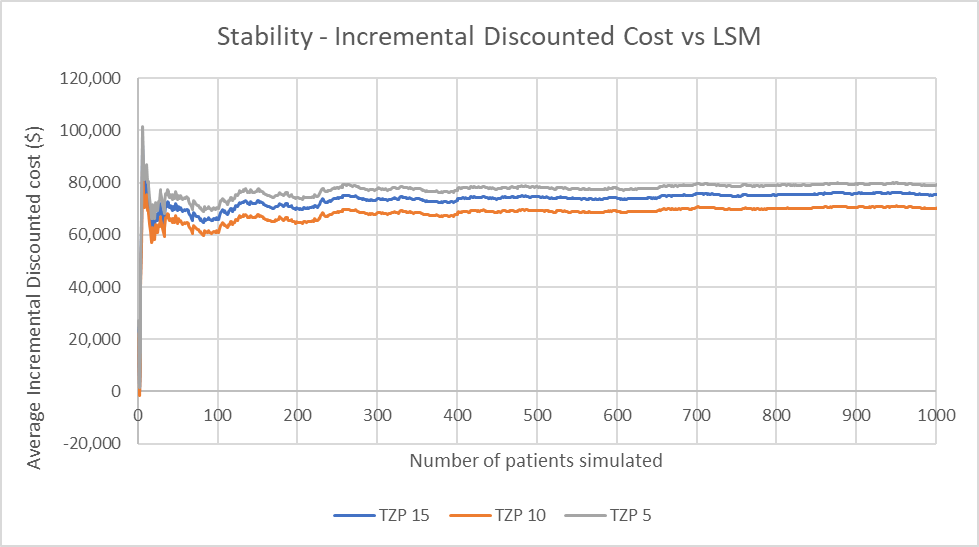


Stability plots show the average incremental outcome on the y-axis, given the number of patients simulated on the x-axis. They are used to assess whether a sufficient number of replications have been run to achieve a stable result, indicated by the presence of a flat line.

*Figure S3. Cumulative Incidence: Deaths
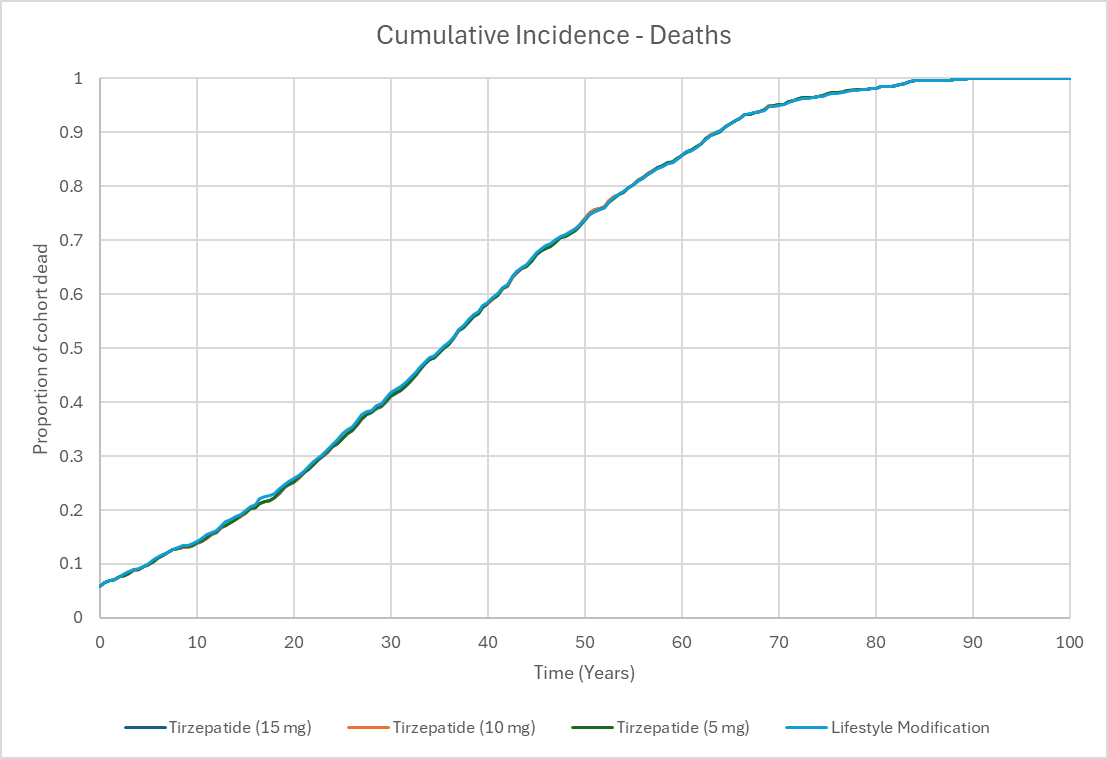
*

*Figure S4. Cumulative Incidence: Type 2 Diabetes*
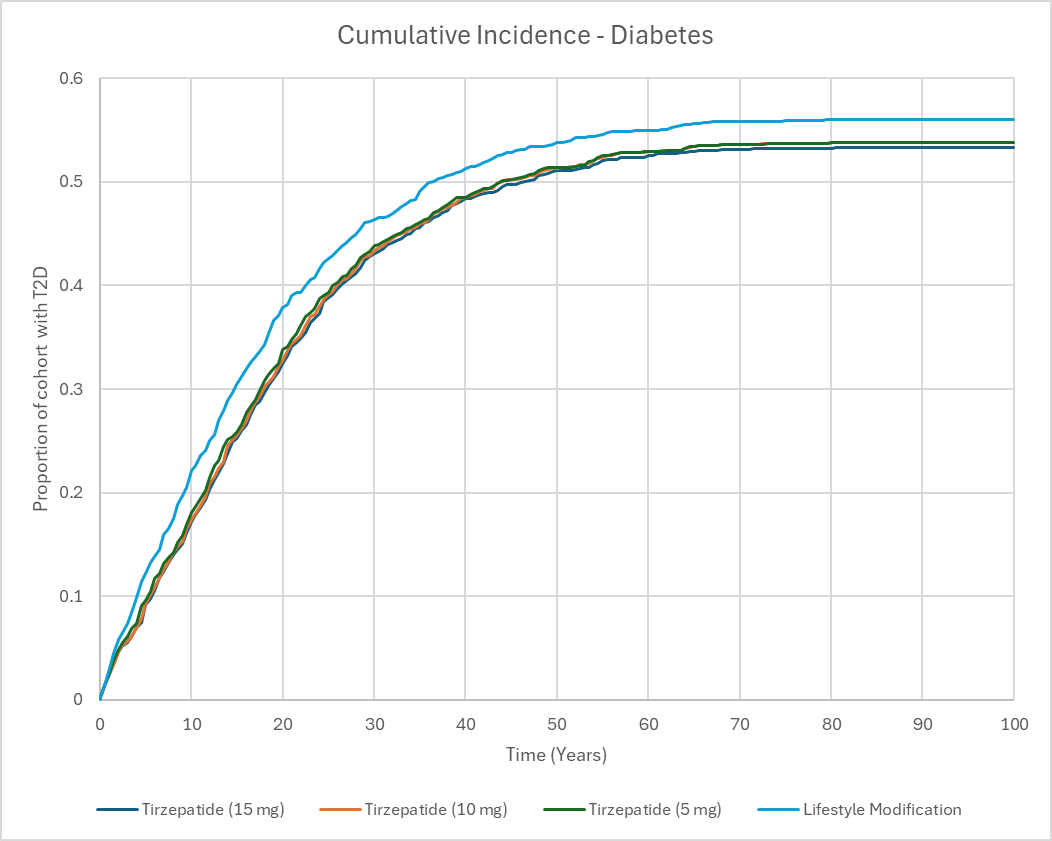


*Figure S5. Cumulative Incidence: Any CV Event*
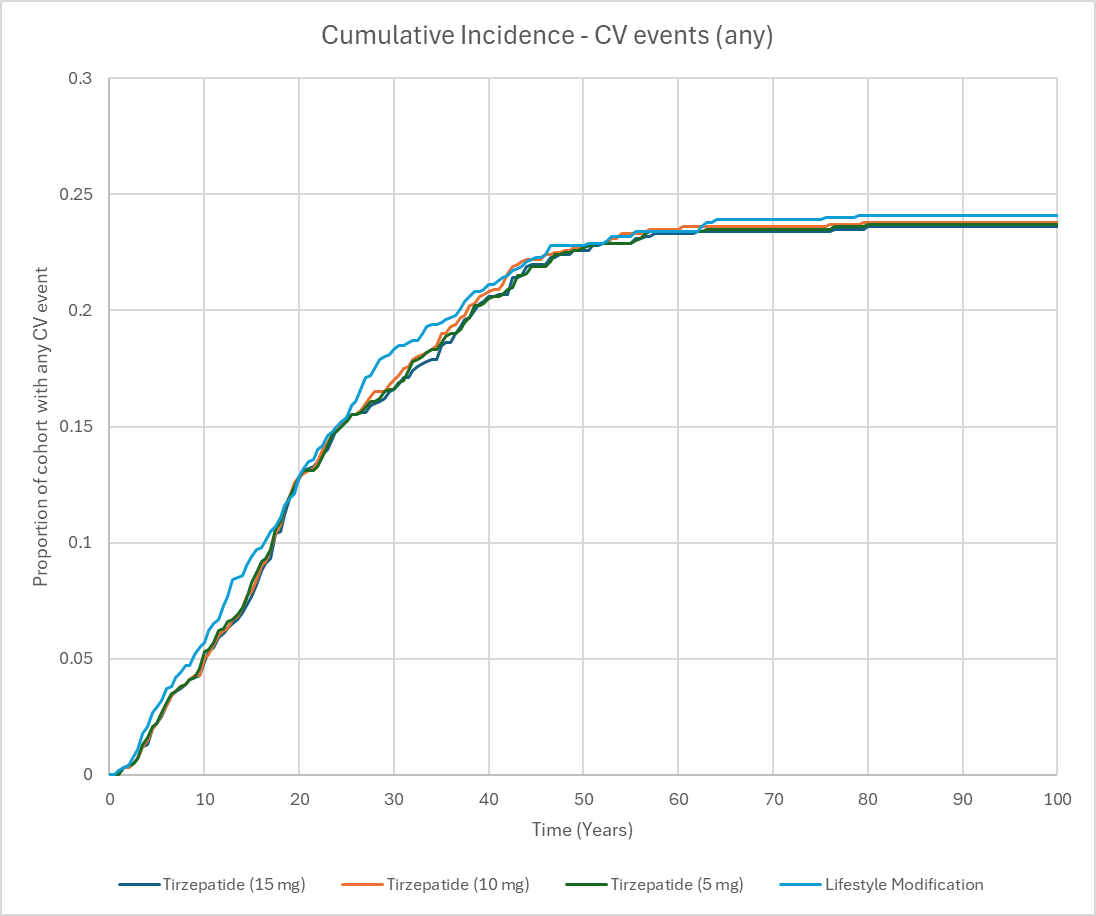


*Figure S6. Stability Plotting: PSA, Incremental LY
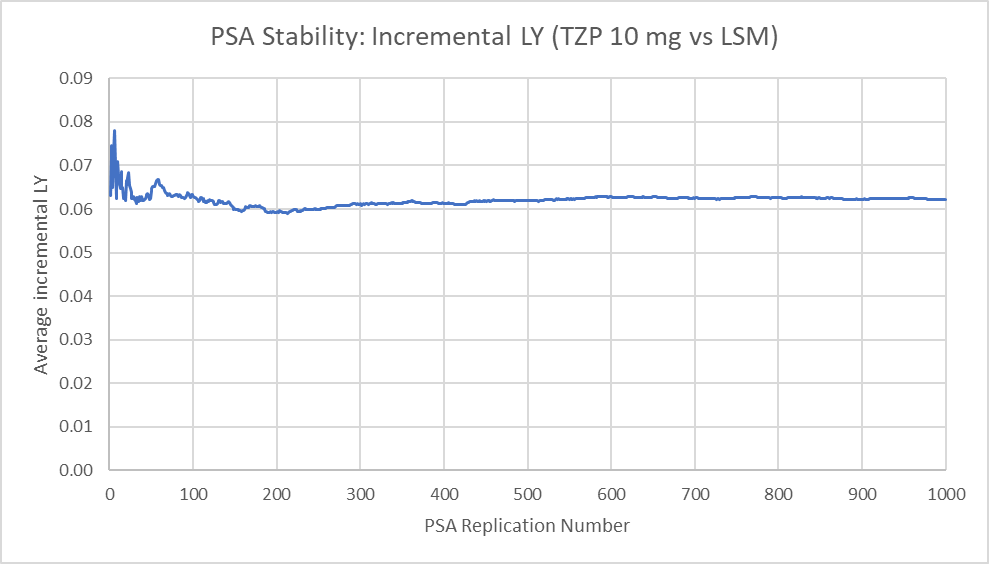
*

*Figure S7. Stability Plotting: PSA, Incremental QALY
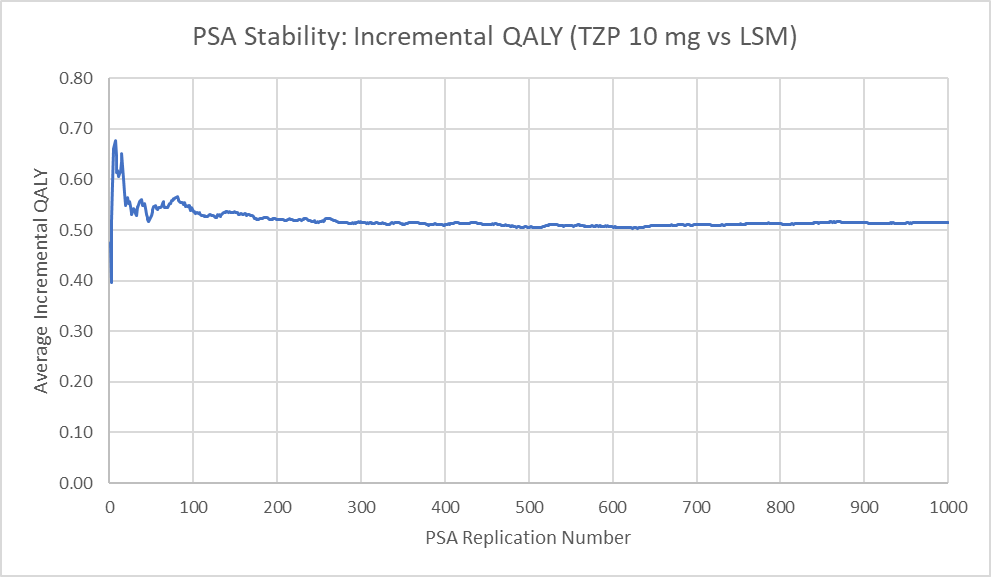
*

*Figure S8. Stability Plotting: PSA, Incremental Costs
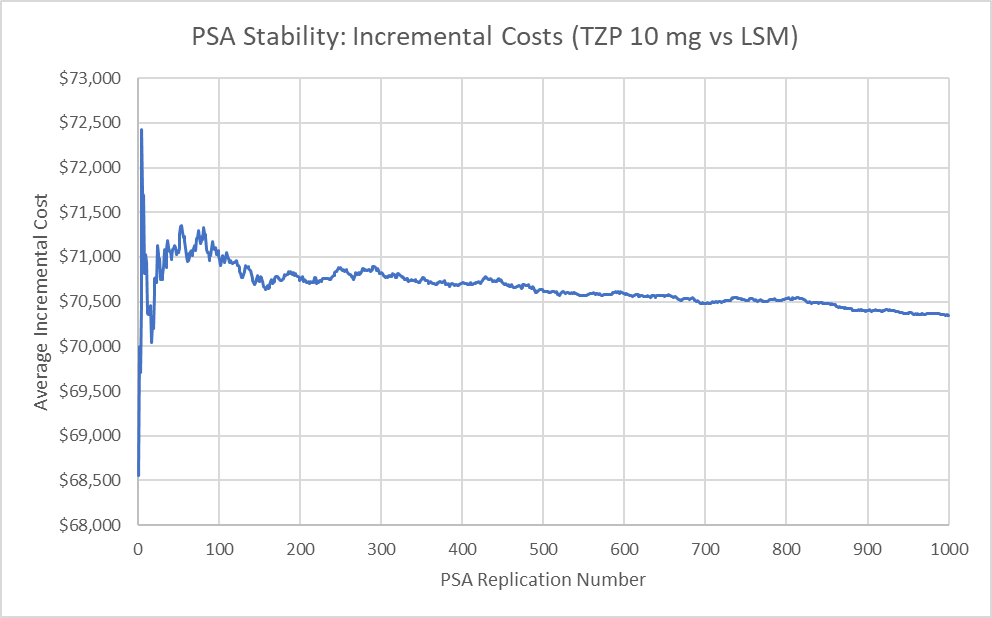
*

*Figure S9. CE Plane – Tirzepatide 10mg vs. LSM*

*
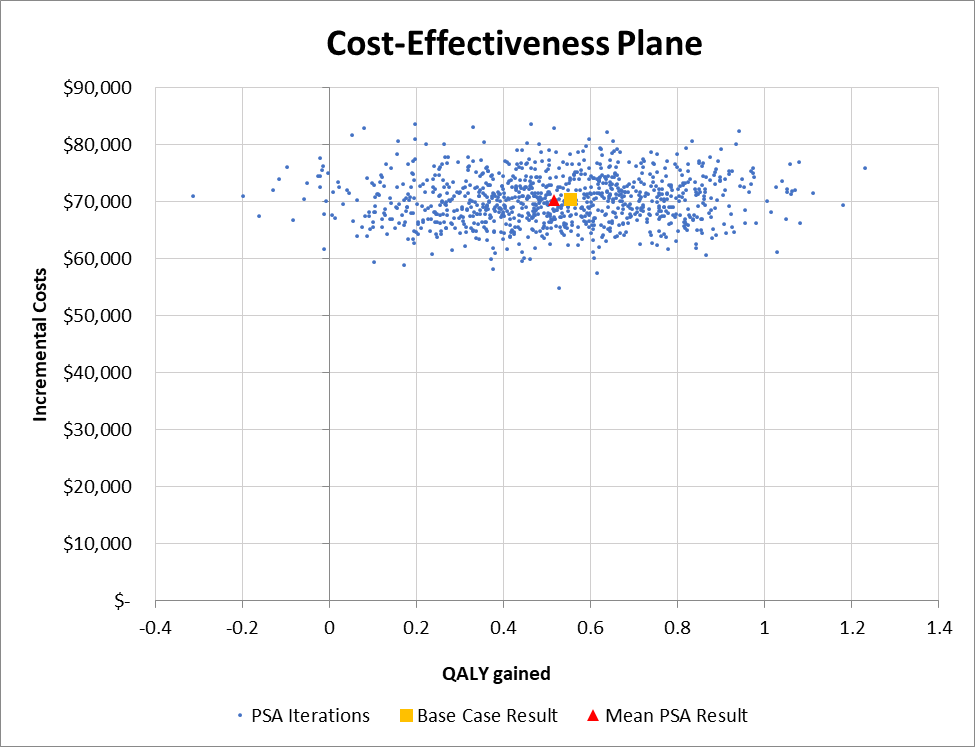
*

*Figure S10. Cost-Effectiveness Acceptability Curve*


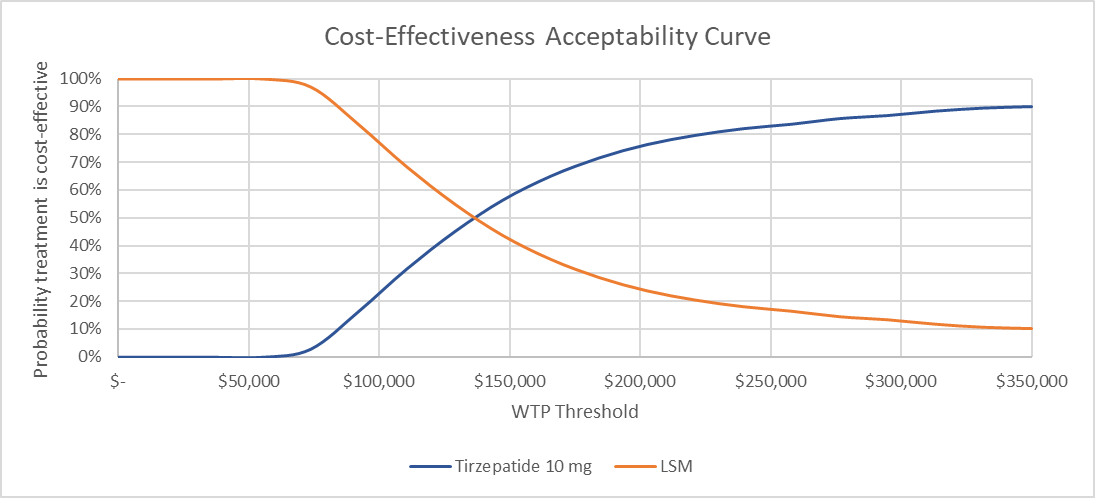


# References

1. Shamai L, Lurix E, Shen M, et al. Association of body mass index and lipid profiles: evaluation of a broad spectrum of body mass index patients including the morbidly obese. *Obes Surg*. 2011;21(1):42-47. doi:10.1007/s11695-010-0170-7

2. Adler C, Schaffrath Rosario A, Diederichs C, Neuhauser HK. Change in the association of body mass index and systolic blood pressure in Germany - national cross-sectional surveys 1998 and 2008-2011. *BMC Public Health*. 2015;15:705. doi:10.1186/s12889-015-2023-8

3. Merative. Merative micromedex RED BOOK. Accessed April 29, 2024. <https://www.micromedexsolutions.com/micromedex2/librarian/CS/A54CAC/ND_PR/evidencexpert/ND_P/evidencexpert/DUPLICATIONSHIELDSYNC/38AE51/ND_PG/evidencexpert/ND_B/evidencexpert/ND_AppProduct/evidencexpert/ND_T/evidencexpert/PFActionId/redbook.FindRedBook?navitem=topRedBook&isToolPage=true>

4. InHealth Professional Services. *2020 Physicians’ Fee and Coding Guide*. 2020.

5. Centers for Medicare & Medicaid Services. Files for FY 2020 final rule and correction notice. Accessed April 29, 2024. <https://www.cms.gov/medicaremedicare-fee-service-paymentacuteinpatientppsacute-inpatient-files-download/files-fy-2020-final-rule-and-correction-notice>

6. Bonafede MM, Johnson BH, Richhariya A, Gandra SR. Medical costs associated with cardiovascular events among high-risk patients with hyperlipidemia. *Clinicoecon Outcomes Res*. 2015;7:337-345. doi:10.2147/CEOR.S76972

7. Urbich M, Globe G, Pantiri K, et al. A systematic review of medical costs associated with heart failure in the USA (2014-2020). *Pharmacoeconomics*. 2020;38(11):1219-1236. doi:10.1007/s40273-020-00952-0

8. Agency for Healthcare Research and Quality. NIS overview. Updated February 1, 2024. Accessed December 1, 2020. <https://hcup-us.ahrq.gov/nisoverview.jsp>

9. Rein DB, Zhang P, Wirth KE, et al. The economic burden of major adult visual disorders in the United States. *Arch Ophthalmol*. 2006;124(12):1754-1760. doi:10.1001/archopht.124.12.1754

10. Honeycutt AA, Segel JE, Zhuo X, Hoerger TJ, Imai K, Williams D. Medical costs of CKD in the Medicare population. *J Am Soc Nephrol*. 2013;24(9):1478-1483. doi:10.1681/ASN.2012040392

11. Boyko EJ, Monteiro-Soares M, Wheeler SGB. Peripheral arterial disease, foot ulcers, lower extremity amputations, and diabetes. In: Cowie CC, Casagrande SS, Menke A, et al., eds. *Diabetes in America*. 3rd ed. 2018. <https://www.ncbi.nlm.nih.gov/pubmed/33651539>

12. An J, Nichols GA, Qian L, et al. Prevalence and incidence of microvascular and macrovascular complications over 15 years among patients with incident type 2 diabetes. *BMJ Open Diabetes Res Care*. 2021;9(1)doi:10.1136/bmjdrc-2020-001847

13. Mariotto AB, Yabroff KR, Shao Y, Feuer EJ, Brown ML. Projections of the cost of cancer care in the United States: 2010-2020. *J Natl Cancer Inst*. 2011;103(2):117-128. doi:10.1093/jnci/djq495

14. Alsumali A, Eguale T, Bairdain S, Samnaliev M. Cost-effectiveness analysis of bariatric surgery for morbid obesity. *Obes Surg*. 2018;28(8):2203-2214. doi:10.1007/s11695-017-3100-0
